# Continuous Adductor Canal Block used for postoperative pain relief after medial Unicondylar Knee Arthroplasty: a randomized, double-blind, placebo-controlled trial

**DOI:** 10.1186/s12871-019-0787-6

**Published:** 2019-06-29

**Authors:** Fei Lan, Yanyan Shen, Yanhui Ma, Guanglei Cao, Nicole Philips, Ting Zhang, Tianlong Wang

**Affiliations:** 1 Department of Anesthesiology Xuanwu Hospital, Capital Medical University, No.45, Changchun Street, Beijing, 100053 China; 2 Department of Anesthesiology, Peking University International Hospital, Beijing, China; 3 Department of Orthopedics Xuanwu Hospital, Capital Medical University, Beijing, China; 4 Department of Critical Care Medicine St. Michael’s Hospital, University of Toronto, Toronto, Canada

**Keywords:** Knee, Arthroplasty, Adductor canal block, Local, Analgesia

## Abstract

**Background:**

Peripheral nerve block and local infiltration analgesia (LIA) provide good analgesia after knee replacement. This study evaluated the additional analgesic efficacy of continuous adductor canal block (ACB) added to single-dose LIA after medial unicondylar knee arthroplasty (UKA). We hypothesized ACB would lower pain scores and facilitate postoperative ambulation.

**Methods:**

Forty-six patients were enrolled into this double-blind, randomized, placebo-controlled trial. UKA was performed and all patients received single-dose LIA intraoperatively. Patients were randomized into two groups: Group RP receiving 0.2% ropivacaine or Group Con receiving normal saline. A flow at 6 mL/h was administered for 48 h through a catheter in the adductor canal. Primary outcome was movement pain score at 24 h using the numeric rating scale (NRS-11). Secondary outcomes included serial postoperative pain scores, rate of patients with NRS>3 at rest and movement within 24 and 48 h postoperatively, time to breakthrough pain, quadriceps motor strength, ambulated distance, catheter related infection and patient satisfaction.

**Results:**

Forty-two patients were analyzed. Pain scores with movement at 24 h postoperatively were significantly lower in Group RP than that in Group Con (3 vs. 5 NRS, P<0.001). Compared with Group Con, breakthrough pain occurred later in Group RP (18.5 vs 10.0 h, *P* = 0.002), serial pain scores at rest and with movement and rate of patients with NRS>3 with movement after surgery were significantly lower. Quadriceps motor strength was equivalent, however, ambulated distance on postoperative day 1 and 2 in Group Con was significant less (19.7 vs 37.3 m, *P* = 0.046; 33.4 vs 59.5 m, *P* = 0.002).

**Conclusions:**

Continuous adductor canal block added to single-dose LIA offered better analgesia and facilitated ambulation without motor weakness after medial UKA.

**Trial registration:**

Clinical Trial Registration: ChiCTR-IOR-16008720; Registered 25 June 2016.

## Background

Similar to total knee arthroplasty (TKA), moderate to severe pain caused by surgical trauma and early functional rehabilitation is anticipated after medial unicondylar knee arthroplasty (UKA) [1]. Optimal pain management, while minimizing analgesia-related complications is imperative, as pain after UKA can largely affect early ambulation, rehabilitation, and discharge [2]. Multimodal analgesic regimens, which include pain medications, local infiltration anesthesia (LIA) and peripheral nerve blocks (PNB), may be the most effective way of managing pain after major joint arthroplasty [3, 4]. While each regimen works well following TKA, femoral nerve blockade (FNB) has traditionally been the gold standard for analgesia [5]. The major disadvantages to FNB include, short duration and muscle strength reduction, and as a result, an alternative method is required [6, 7]. Recently, adductor canal block (ACB) has been suggested to be an alternative to FNB and has been shown to provide equivalent analgesia, while preserving quadriceps motor strength [8–10] and facilitating ambulation [11, 12].

The anterior cutaneous branches of the femoral nerve, the saphenous nerve, and branches of the obturator nerve travel through the adductor canal in the medial part of the thigh and innervate the surgical area involved in a medial UKA [13–15]. Previous studies focusing on TKA have suggested that single shot or continuous ACB added to a single-dose LIA can decrease postoperative pain and opioid consumption [16, 17]. Only one study reported that a single shot ACB given preoperatively may provide equivalent analgesia after medial UKA when compared with psoas compartment block [18]. Furthermore, no studies have reported the effect of continuous ACB combined with single-dose local infiltration analgesia (LIA) as a multimodal analgesic regimen after medial UKA.

Therefore, this prospective, randomized, double-blind, placebo-controlled trial compared the effects of continuous ACB added to an intraoperative single-dose LIA after medial UKA. We hypothesized that a continuous infusion ACB, in addition to LIA, would lower pain scores with movement at 24 h after surgery (primary outcome). We also hypothesized that this would improve serial pain scores, preserve quadriceps motor strength during physiotherapy, and facilitate ambulation within 48 h after surgery (secondary outcomes).

## Methods

### Ethics and registration

Approval was obtained from the Institutional Review Board of Xuanwu Hospital, Capital Medical University, code: 2017(074). The study was prospectively registered at Chictr.org.cn (code: ChiCTR-IOR-16008720) on June 25, 2016, and written informed consent was obtained from all participants before enrollment.

### Patient inclusion and exclusion criteria

This prospective, randomized, double-blind, placebo-controlled trial was conducted from March 2017 to February 2018. Patients between 55 and 75 years of age were included if they were scheduled for medial UKA under spinal anesthesia (SA) with the American Association of Anesthesiologists (ASA) physical status of I-II. Patients were excluded if they had a history of opioid addiction, allergy to any of the study medications, a contraindication to ACB (peripheral neuropathy and infection at the procedure site) and/or a contraindication to SA (coagulopathy and recent anti-coagulant medication use).

### Randomization and blinding

Randomization was carried out using a computer-generated randomization list. Patients were randomized into two groups; one receiving 0.2% ropivacaine (Group RP), and a control receiving normal saline (Group Con) via the adductor canal. Each patient received a consecutive study number and treatment assigned by the randomization list. The list was stored and only two nurses, who prepared the study medications were allowed access. They had no interaction with the patients. All other medical personnel, participants and outcome assessors were blinded to the interventions.

### Administration of anesthesia and surgical procedure

All patients received spinal anesthesia through a median or para- median approach using a 26 or 27 G Whitacre needle with 2.0 ml 0.5% bupivacaine at the L3/4. Sedation with propofol and fluid therapy were administered intraoperatively by an anesthesiologist. Surgical technique was identical for all patients and all procedures were done in a bloodless field by use of a femoral tourniquet. Unless contraindicated, all patients were given oral preoperative multimodal analgesic medications including 400 mg celecoxib and 1000 mg acetaminophen, according to the patients’ weight. Ondansetron 4 mg intravenous injections were administered prophylactically to prevent postoperative nausea and vomiting.

### LIA and continuous ACB

All patients received LIA, consisting of a total of 100 ml 0.2% ropivacaine, 10 mg oxycodone and 0.5 mg adrenaline. All solutions were prepared under aseptic conditions. This is routinely performed by the surgeon for all medial UKAs before prosthesis implantation and wound closure. Using a similar method described previously [19, 20], 40 mL of the mixture was injected into the posterior capsule and the medial and lateral ligaments before inserting the components, Another 30 ml was injected into the anterior capsule, the synovium and retinacular tissues after insertion of the implants. The remaining mixture was infiltrated into the infrapatellar fat pad and the subcutaneous tissues before the closure of wound.

Upon completion of the surgery, patients were transferred to the post-anesthesia care unit, where standard monitoring was provided and continuous ACB was performed before spinal anesthesia had worn off.

A total of 300 mL (280 ml for infusion and a 20 ml bolus injection) of study solution, either 0.2% ropivacaine or normal saline, was prepared by either of the two unblinded nurses immediately after surgery.

The adductor canal was identified at mid-thigh level under ultrasound guidance and an 18-gauge Pajunk needle was inserted into the canal. A 20 mL bolus of the study drug (0.2% ropivacaine or normal saline) was administered. A bolus injection of 20 mL is required to fill the canal without risking retrograde flow to the femoral triangle [13, 21]. A 22-gauge Pajunk catheter was then placed through the needle and advanced a further 5 cm into the canal. The position was confirmed by ultrasound with a 2–3 mL injection of normal saline. Four hours after bolus injection, a continuous infusion by an electronic pump was activated at 6 mL/h for 48 h. If signs of irritation, allergy, or infection were observed at the catheter site, the intervention was stopped immediately and the patient was excluded from the study. The catheter was removed on postoperative day 2 following the afternoon physiotherapy session.

All patients received a multimodal pain regimen postoperatively: oral acetaminophen 1000 mg and oral celecoxib 200 mg every 12 h. In addition, rescue analgesics were available with oral fast-release oxycodone ≤10 mg every 4 h or as needed. If intolerance of oral medication, the patient was given, IV morphine 2.5 mg every 1 hour or as needed.

### Outcome measures

Demographic data were collected preoperatively. The preoperative maximum range of knee motion was assessed. Surgical and spinal block duration, and the length of surgical incision were also recorded. Research personnel blinded to group assignment performed all pre- and postoperative assessments and data collection.

### Primary outcome

The primary end point was pain scores with active knee flexion in the operated knee at 24 h after surgery. At the time of the assessment, patients were instructed to record their pain on NRS-11 [22]. The numeric rating scale (NRS) is a tool that allows patients to express their perceived pain, where 0 indicates no pain and 10 indicates the worst possible pain. The NRS-11 was explained to patients in great detail preoperatively.

### Secondary outcomes

Pain scores using the NRS-11 and the numbers of patients with NRS>3 at 8, 12, 24, and 48 h after surgery were measured at rest and with movement. Additionally, the investigators recorded the first time point of postoperative pain at rest greater than 3 (NRS > 3), known as breakthrough pain. Opioid consumption during 0–24 h and 24–48 h postoperatively was retrieved from the electronic medical record and oral oxycodone converted to IV morphine equivalents for analysis [23, 24]. As for the ambulation ability assessments, patients were mobilized at least twice on postoperative day (POD) 1 and 2 with physical therapy assistance. During each physical therapy session, patients were asked to ambulate as far as possible. The total ambulated distance, measured in meters, was recorded by blinded outcome assessors. Quadriceps muscle strength was assessed at 4, 8, 12, 24, and 48 h postoperatively by blinded research personnel. Patients were asked to hold the affected limb up with the knee extended against resistance of the examiner and assigned a number using the manual muscle testing (MMT) grading scale (0 = no contraction, 1 = flicker of contraction, 2 = active movement with gravity eliminated, 3 = active movement against gravity but not resistance, 4 = active movement against gravity and some resistance and 5 = normal strength). Moreover, nerve block and catheter related complications and patient satisfaction were also assessed at 24 and 48 h postoperatively and all patients were asked to give a dichotomous verbal assessment (“Satisfied” or “Unsatisfied”) of the quality of analgesia.

### Statistical analysis

Statistical analysis was performed using IBM SPSS 20 (IBM Corporation, Armonk, New York). According to a pilot study of 12 patients receiving LIA without continuous ACB in our hospital, the mean pain score on movement at 24 h after surgery was NRS = 4.8 [SD, 2.6]. This value has been reported by other, similar studies [19, 25]. Our study intervention was modelled after Andersen (2013), who used combined analgesia after TKA [26]. As a result of combined analgesia, we expected to see a decrease of ≥2 NRS points on movement at 24 h postoperatively in the treatment group. A sample size of 38 patients (19 in each group) was required for a type I error of 0.05 and a power of 90%. Taking into account a potential dropout rate of 20%, we aimed to recruit 46 patients in this study. Unpaired t-tests were used for the statistical analyses and continuous variables are presented as mean (SD). Ordinal and non-normally distributed variables are expressed as median (range), and the Mann-Whitney U test was applied. Dichotomous data (gender, rate of patients with NRS>3 and patient satisfaction) were analyzed using the chi–square test or Fisher’s exact test. A *p* < 0.05 was considered to be statistically significant.

## Results

**Fig. 1:**
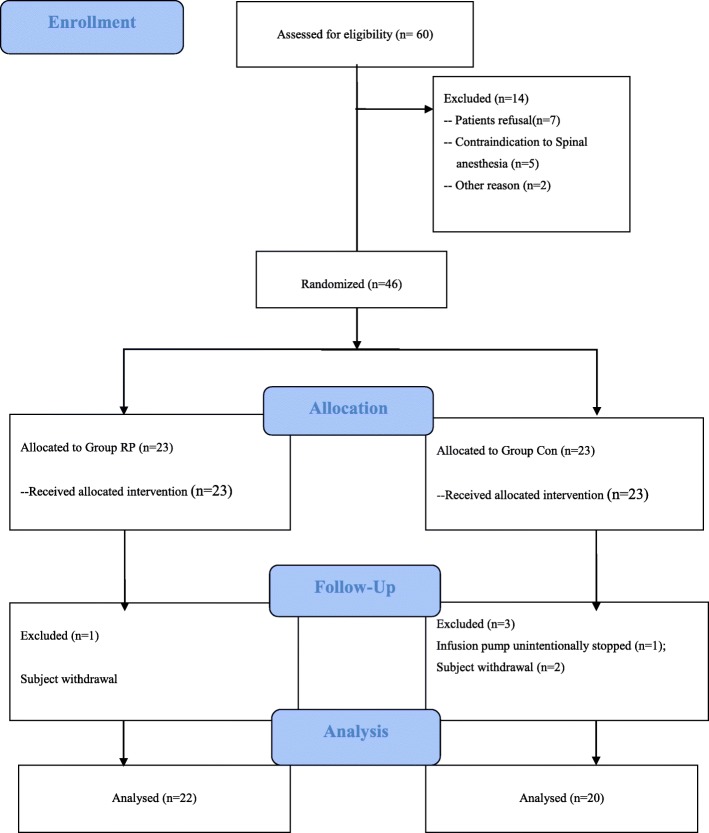
Flow chart of the study

Sixty patients were approached for participation in this study. Forty-six patients were finally included and randomized to the treatment group or control group. Forty-two patients completed the study and were analyzed for outcomes. Four subjects were excluded due to protocol violations (Fig. 1). Of the 4 subjects excluded, 3 subjects from both groups requested to withdraw from the study, and 1 subject from Group Con had pump failure during the night. Preoperative measurements and demographic data were similar between groups. Moreover, there was no difference between groups with respect to surgery and spinal block durations, or length of surgical incision (*P* > 0.05, Table 1).

**Table 1:** Demographics and baseline characteristics

	Group RP (*n* = 22)	Group Con (*n* = 20)	*P* value
Age, (y)	66.1 ± 7.2	67.9 ± 6.5	0.397
Weight, (kg)	71.9 ± 9.6	67.3 ± 10.9	0.146
Height, (cm)	162.27 ± 4.92	155.85 ± 4.93	0.221
Body mass index, (kg/m2)	27.3 ± 3.7	27.6 ± 3.8	0.829
Sex, (male)	5	4	0.578
Duration of surgery, (min)	136 ± 22	124 ± 17	0.058
Duration of spinal block, (min)	143 ± 7	140 ± 9	0.215
Surgical incision length, (cm)	13.4 ± 3.0	12.0 ± 3.0	0.097
Range of motion before surgery, (degree)	102 ± 13	102 ± 16	0.667

Values are shown as mean ± SD

**Fig. 2:**
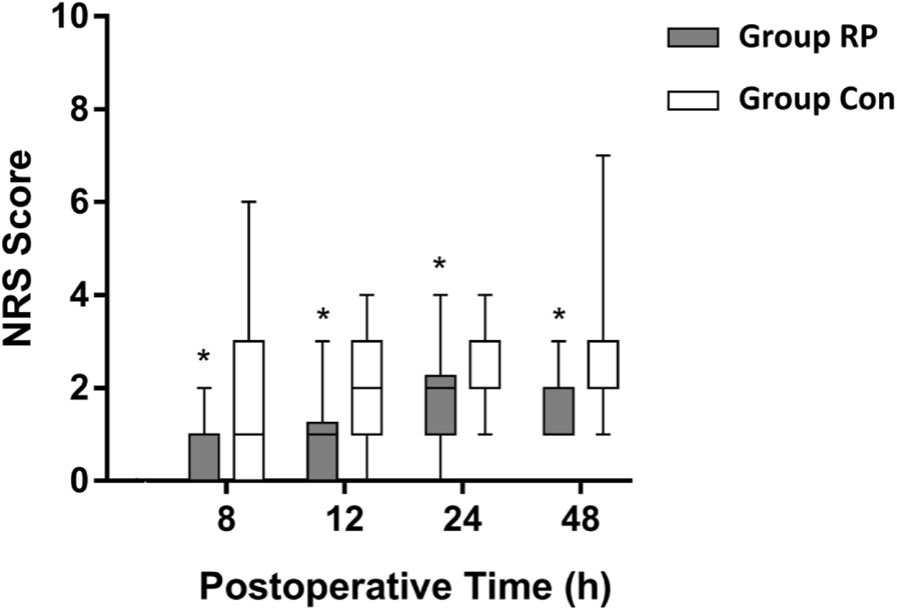
Pain assessment at different time points postoperatively at rest. Data are expressed as median (horizontal bar) with 25th–75th (box) percentile and minimum to maximum (whiskers). *P < 0.05

**Fig. 3:**
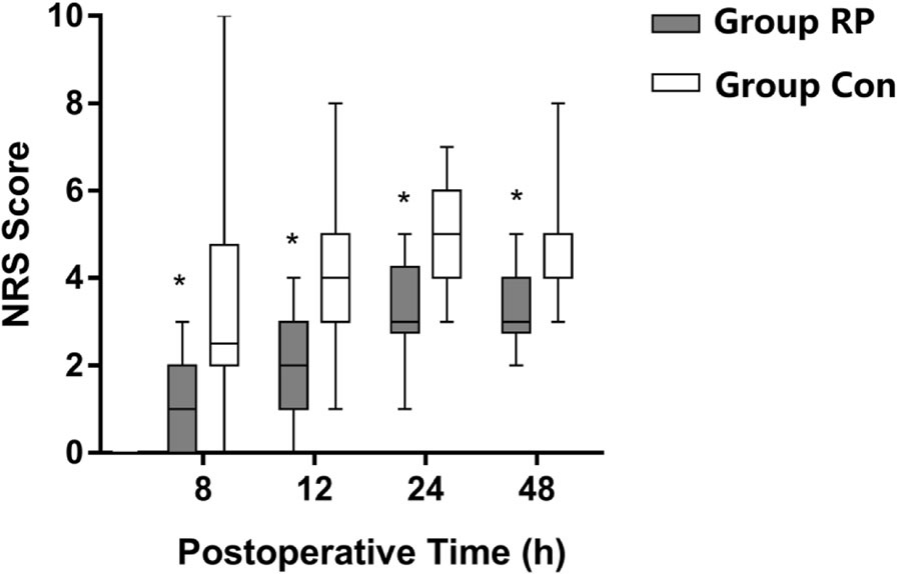
Pain assessment at different time points postoperatively with movement. Data are expressed as median (horizontal bar) with 25th -75th (box) percentile and minimum to maximum (whiskers). *P < 0.05

The primary end point of pain scores with active knee flexion in the operated knee at 24 h after surgery was significantly reduced in Group RP compared with Group Con (3 [IQR, 2.75–4.25] vs 5 [IQR, 4–6], *P*<0.001) (Table 2). Furthermore, time until breakthrough pain (NRS > 3) was significantly longer in Group RP than that in Group Con (18.5 [IQR, 4–46] hours vs 10.0 [IQR, 3–24] hours, *P* = 0.002) (Table 2). In addition, NRS pain scores at rest and with movement at 8, 12, 24 and 48 h after surgery (Figs. 2 and 3), and rate of patients with NRS>3 with movement within 24 and 48 h postoperatively were significantly lower in Group RP than in Group Con (Table 2)(*P* < 0.05). As for the consumption of IV morphine, there was no significant difference between groups 0–24 h after surgery. However, Group RP consumed significantly less IV morphine at 24–48 h postoperatively compared to Group Con (15.64 ± 10.53 mg vs 27.15 ± 21.46 mg, *p* = 0.039) (Table 3).

**Table 2:** Primary endpoint, percentage of patients with NRS pain score>3 within 24 and 48 h postoperatively, first time point of breakthrough pain and ambulated distance postoperatively

	Group RP *n* = 22	Group Con *n* = 20	*p*-value
NRS durimg active knee flexion at 24 h postoperatively	3 (2–4)	5 (4–6)	<0.001
Patients with NRS>3 at rest. No. (%)			
within 24 h postoperatively	2 (3)	6 (10)	0.150
within 48 h postoperatively	2 (2)	8 (10)	0.049
Patients with NRS>3 with movement. No. (%)			
within 24 h postoperatively	8 (12)	39 (65)	<0.001
within 48 h postoperatively	17 (19)	54 (68)	<0.001
Time to breakthrough pain (NRS > 3), (hours)	10 (3–24)	18 (4–46)	0.002
Ambulated distance on POD 1, (meters)	37.3 ± 32.2	19.7 ± 22.1	0.046
Ambulated distance on POD 2, (meters)	59.5 ± 28.3	33.4 ± 20.8	0.002

Data are shown as counts, median (interquartile range) or a mean ± SD; NRS = Numeric rating scale (for assessment of pain intensity)

**Table 3:** Morphine consumption, patient satisfaction and catheter related infection after surgery

	Group RP (*n* = 22)	Group Con (*n* = 20)	*P* value
IV morphine consumption, (mg)			
0–24 h postoperatively	13.82 ± 5.50	17.8 ± 7.41	0.063
24–48 h postoperatively	15.64 ± 10.53	27.15 ± 21.46	0.039
Satisfied patients, No. (%)			
24 h postoperatively	19 (86)	17 (85)	0.617
48 h postoperatively	18 (81)	15 (75)	0.437
Nerve blocking and catheter related complications, No. (%)	0	0	–

Values are shown as mean ± SD or frequency (%)

**Fig. 4:**
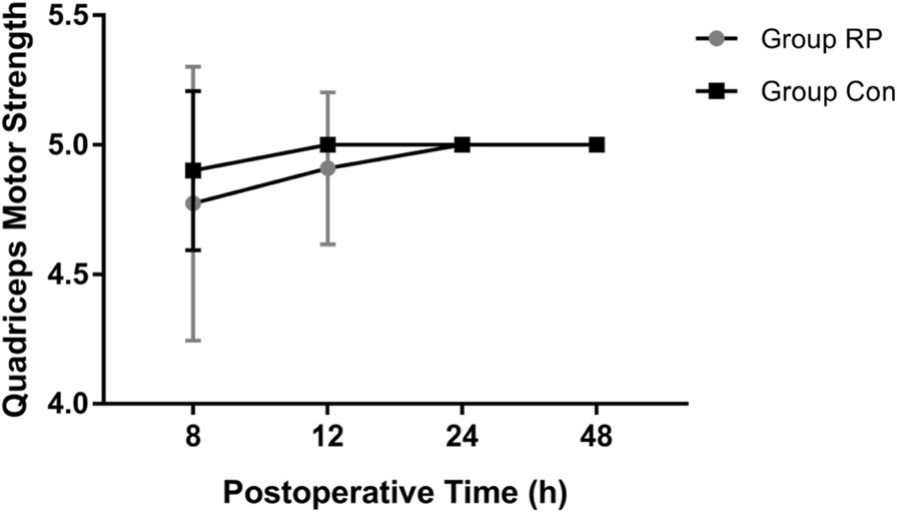
Quadriceps muscle strength assessment postoperatively. Data are expressed as mean (SD)

There was no difference between groups for quadriceps muscle strength assessed at different postoperative time points (*P* > 0.05) (Fig. 4). However, the treatment group showed a statistically significant improvement in maximum distance ambulated compared with that of the control group on POD 1 and 2: (37.3 ± 32.2 vs 19.7 ± 22.1, *P* = 0.046; 59.5 ± 28.3 vs 33.4 ± 20.8, *P* = 0.002) (Table 2).

In addition, there was no nerve block and catheter related complications to be reported in either groups, and no difference was found in patient analgesia satisfaction at 24 and 48 h postoperatively (86% vs 85%, *P* = 0.617; 81% vs 75%, *P* = 0.437) (Table 3).

## Discussion

Our findings demonstrate that the addition of a continuous ACB to single-dose of LIA after medial UKA significantly reduced pain scores with knee movement at 24 h after surgery. This result is strengthened by the fact that the time until breakthrough pain was significantly longer in Group RP. Furthermore, better pain relief was demonstrated by the fact that patients in the treatment group were better able to ambulate on POD 1 and 2.

Previous studies have demonstrated improved pain relief and decreased opioid consumption in patients receiving LIA after knee arthroplasty [27, 28]. However, periarticular infiltration analgesic regimens that infiltrate anterior, medial, and posterior compartments of the knee are reported to only last 6 to 12 h [29, 30], which is consistent with our observation from the time until breakthrough pain. Femoral nerve block when applied as part of multimodal analgesic management for patients undergoing TKA has been reported to decrease opioid consumption and lower postoperative pain scores [5]. Despite the improved analgesic outcomes, prolonged motor block and quadriceps weakness from femoral nerve block inhibit “fast track” rehabilitation [31, 32]. NRS pain scores on movement (knee flexion) at 24 h was chosen as the primary outcome in this study. Assessing pain at this time point is important for determining adequate analgesia for starting physical therapy, as the first physical therapy session was initiated 24 h postoperatively. In addition, previous studies have suggested that movement pain is more important than rest pain [33]. In this study, the duration of spinal anesthesia with 10 mg bupivacaine was approximately 15 min more than surgical duration in Group Con and 6 min more than surgical duration in Group RP. Moreover, the first pain assessment was initiated at 8 h postoperatively. Therefore, spinal anesthesia, which impairs the quality of clinical assessment could be ignored.

Our results support the addition of a continuous ACB to a single-dose LIA after medial UKA to supply sufficient analgesia, especially with movement, and help with ambulation after the day of surgery. Simple time-by-time comparisons for the repeated pain measurements, strongly inflated the type-I error. As a result, we transformed the data into number of relevant events (NRS>3) and compared the rates after surgery. We found the major difference of pain scores between groups occurred during movement. Similar to this study, previous studies had suggested that continuous ACB combined with single dose LIA can reduce pain scores (at rest and with movement) and opioid consumption after total knee replacement [26, 34]. Andersen et al. reported that saphenous nerve block with single-dose LIA offered better pain relief on the day of surgery than LIA alone after TKA, but no validated physiotherapy testing was used to compare the groups in that study. Conversely, Gudmundsdottir and Franklin reported that there is no pain related benefit to be acquired from adding an ACB to a single-dose LIA during physiotherapy session on POD 1 after TKA [17]. The main reason our results differ relates to the type of knee surgery itself. Total knee arthroplasty is invasive and more painful following surgery, leading to the need for more potent pain relief postoperatively. UKA is characterized by short incisions, less osteotomy and is capable of rapid recovery [35] . However pain is still an important issue in early postoperative functional rehabilitation [1, 36], which was consistent with what we found when comparing the rate of patients with NRS>3 with movement in control group. In this study, the mean surgical incision in both groups was over 12 cm long, similar to conventional surgery. However, it has been reported that even with minimally invasive surgery of UKA (an 8 to 10 cm-long medial parapatellar skin incision), pain scores and functional outcomes were not improve by using LIA alone [37]. Considering the surgical area of nerve innervation in medial UKA, continuous ACB may be uniquely suited to provide postoperative analgesia. Therefore, it is readily explained that continuous ACB plus single shot LIA can reduce pain scores at rest and with movement after surgery, and facilitate ambulation as shown in this study.

Patient satisfaction was assessed as “satisfied” or “unsatisfied” at 24 and 48 h postoperatively. Essving (2009) reported that pain scores at rest and with movement were acceptable for patients who underwent medial UKA with intra-articular LIA combined with perioperative oral analgesics within 24 h postoperatively [19]. This is similar to the control group in this study. Therefore we are not surprised that there is no difference in patient satisfaction at 24 h after surgery. Furthermore, there was no difference in IV rescue morphine consumption during 0-24 h postoperatively, although NRS pain scores within 24 h after surgery were significantly lower in Group RP. However, during the 24–48 h postoperative period, intra-articular LIA had completely worn off, leading to an increase in overall pain scores seen in Group Con and likely had negative effects on physiotherapy after post-operative day 1. Increased pain likely led to the increase in IV rescue morphine consumption seen in Group Con during this time period. Therefore, patient satisfaction at 48 h postoperatively in Group Con was lower than in Group RP, although there was no statistical difference.

Motor block caused by peripheral nerve block in the lower extremities is a well-known adverse effect that compromises rehabilitation and even causes a risk of falling [38, 39]. There are case reports to suggest that ACB can affect quadriceps muscle strength, which can limit ambulation abilities [40, 41], however, this seems to be rare. In our study, at 48 h after surgery, there was no difference in quadriceps muscle strength between groups, which likely facilitated patients’ early ambulation. From pain evaluation scores at different time points after surgery, it is not difficult to understand why the ambulated distance of patients in the treatment group was much longer compared with the control group. Pain was better managed during the first 48 h after surgery and the quadriceps muscle strength was well maintained.

The use of an invasive placebo may raise ethical concerns for some readers. Although it has been debated that invasive placebos are not consistent with ethical practice [42, 43], there is no consensus on the issue within the research community, nor are there uniform standards between ethics committees. The current study was approved by the Institutional Review Board of Xuanwu Hospital, Capital Medical University and all study participants provided informed consent. We assigned blind investigators to assess complications of nerve block in both groups. No patient in either group experienced temporary or permanent complications from the invasive placebo or treatment.

Although there are limitations to a continuous catheter approach [44, 45], such as patients’ unintentional catheter removal, continuous ACB can provide a more prolonged analgesic effect compared with the single-dose method, facilitating rehabilitation on POD 1 and 2. In addition, there was no catheter related complications in either group, and no patient complained of the inconvenience of a portable infusion device.

In this study, the initial dose of ropivacaine for LIA was less than the maximal dose (225 mg) indicated by drug label [46], however, when combined with ACB bolus, the total dose (240 mg) of ropivacaine was slightly higher than recommended. However, previous studies have shown that injecting a much higher dose of ropivacaine in intra-articular LIA, than used in this study, is safe, with plasma levels below systemic toxic threshold [47–50]. Moreover, there was a 60 min interval between injections, which reduced plasma levels. This procedure is considered safe, while also aiming to maximize the duration of the block as safely as possible.

There are several limitations to this study. In order to guarantee all staff and study participants were blinded to the treatment group, we did not assess the success rate of the block. In that way, we cannot confirm that the blocks were all functioning accurately. However, Saranteas et al. has shown about 95% success rate of ACB using a similar approach [44]. In addition, no professional physiotherapists took part in this study, resulting in the inability to record ambulation ability. However, the strengths of our study include, effective randomization, the successful blinding process, and consistent management in standardizing the pre- and postoperative medication. It also was sufficiently powered for the primary end point. Finally, we did not measure total and free plasma concentrations of ropivacaine following LIA and ACB. This would have allowed us to be certain that systemic toxic thresholds were not reached. Although these values were not measured, patients were monitored closely for signs of toxic symptoms which no patient experienced.

## Conclusions

This study suggests that continuous ACB added to single-dose LIA provides sufficient pain treatment after medial UKA and promotes early ambulation. Further studies are needed to address the additional effects that ACB provides to LIA on the day of surgery with a primary focus on ambulation abilities.

## Data Availability

The datasets used and/or analysed during the current study are available from the corresponding author on reasonable request.

## Abbreviations

ACB: Adductor Canal Block
ASA: American Association of Anesthesiologists
FNB: Femoral Nerve Blockade
LIA: Local Infiltration Anesthesia
MMT: Manual Muscle Testing
NRS-11: Numeric Rating Scale
PNB: Peripheral Nerve Blocks
SA: Spinal Anesthesia
TKA: Total Knee Arthroplasty
UKA: Unicondylar Knee Arthroplasty
